# The Cage Treatment of Superficial Wounds

**Published:** 1899-11-18

**Authors:** 


### THE CAGE TREATMENT OF SUPERFICIAL WOUNDS.

Dr. Archdall Reid[1] draws attention to the much better behaviour of surface wounds in the lower animals, such as dogs, as compared with the manner of their healing in man, "especially when the former has not and the latter has the advantage of experienced surgical aid," and he attributes the slow convalescence in man to mistaken efforts on the part of the surgeon. He says that if we insert a piece of the best dressings under the skin violent inflammation ensues. "Usually we have suppuration, which continues until the foreign body is voided in the discharges. . . . But when the skin is destroyed, as in the case of a burn, to these very subcutaneous or subcuticular tissues, which are so intolerant of foreign bodies, we are in the habit of applying foreign bodies—that is, surgical dressings. Surely no more unscientific procedure can well be conceived." He, therefore, advocates the plan of protecting the wound by a wire cage, so arranged as to rest upon the skin clear of the edges of the wound, and then covering this cage with moist dressings. In the case of a limb a complete cylinder of wire network can be made, in two parts, by which the portion of the limb affected can be surrounded. In any case the principle is to thoroughly cleanse the wound, to protect the raw or granulating surface, by aid of the cage, from all contact with dressings or anything else except air, and then to cover up both wound and cage with such dressings as may be necessary to maintain moisture and keep out the dirt. We have no doubt—in fact, we know—that under this method of treatment many superficial wounds do extremely well. At the same time we are hardly prepared to accept Dr. Reid's picture of the healing of wounds under the methods in more general use. We are told that, with the "cage treatment," "the sufferer no longer feels a gradually increasing sensation of heat, pain, and discomfort as the discharges accumulate beneath the sopping dressings, nor does he behold the nauseating mess usually found when the dressings are removed." But this is hardly the best that aseptic surgery can do for us.

[1] Brit. Med. Jour., Oct. 28.

